# Tongue-tie diagnosis using the Lingual frenulum in newborn infants (LINNE) -scoring: A validation study

**DOI:** 10.1371/journal.pone.0338491

**Published:** 2025-12-09

**Authors:** Anu Lehtinen, Venla Lohi, Stiina Aitamurto, Outi Aikio

**Affiliations:** 1 Department of Pediatrics and Adolescent Medicine, Oulu University Hospital, Oulu, Finland; 2 Department of Otorhinolaryngology, Oulu University Hospital, Oulu, Finland; 3 Medical Research Center, Oulu University Hospital, Oulu, Finland; 4 Research Unit of Clinical Medicine, University of Oulu, Oulu, Finland; University of Oklahoma: The University of Oklahoma, UNITED STATES OF AMERICA

## Abstract

**Background:**

Ankyloglossia may restrict newborn infant’s tongue movements, complicating breastfeeding. However, due to lacking evidence-based guidelines, patient selection for early frenotomy has remained a challenge. The present prospective, observation study validated a scoring aimed to detect infants who required early tongue-tie treatment. It is a part of the Lingual Frenulum in Newborn Infants (LINNE) project, investigating tongue-tie epidemiology along with a randomized treatment trial.

**Methods:**

Healthy mother-infant-dyads were assessed by independent and mutually blinded study physicians and midwives. They used the LINNE scoring, including: 1. Infant examination using a picture assessment tool for tongue-tie in breastfed babies (TABBY); 2. Maternal breastfeeding experience scoring (MBES), a psychometric testing on acute breastfeeding symptoms; 3. Three anamnestic factors associated with tongue-ties and breastfeeding problems. Validation tests were conducted using the Cronbach alpha for the whole LINNE scoring, and for the subscorings: 1. TABBY: areas under the curve (AUC), multi-rater Fleiss kappa; 2. MBES: content and construct validity and responsiveness tests; 3. Anamnesis: accuracy studies for diagnostic tests.

**Results:**

Of the studied 556 mother-infant-dyads, 72 (12.9%) infants fulfilled the early tongue-tie treatment criteria. The overall LINNE scoring’s internal consistency was acceptable (alpha = 0.723). The pooled TABBY scoring (n = 1094) accurately detected the need for treatment (AUC = 0.914, 95%CI 0.891–0.938) with moderate inter-rater agreement (kappa = 0.441, 95%CI 0.406–0.477). MBES responsiveness, content, and construct validities reached the required levels. Anamnestic factors had high specificity but fair sensibility for the tongue-tie causing breastfeeding symptoms.

**Conclusion:**

The LINNE scoring demonstrated moderate inter-rater agreement; however, the accuracy of the TABBY tool was very good with AUC 0.9 for ankyloglossia needing early treatment.

## Introduction

The World Health Organization and many national health authorities recommend exclusive breastfeeding as the first-line infant nutrition, up to six months of age [1]. Infants’ oral cavity anomalies, such as ankyloglossia, or tongue-tie, may however cause restrictions on tongue movements and complicate breastfeeding [2–6]. It may involuntarily disturb infant nutrition and growth, change the positive interaction between the mother and child, and subsequently escalate to problems even later in life [7]. The diagnosis of tongue-tie has often based on appearance [8].However, according to some analyses, up to 50% of tight looking frenulums never caused breastfeeding difficulties [9]. Tongue-tie treatments, mostly frenotomy, reduced breastfeeding symptoms [3,10–13]. Despite efforts, the lack of consensus on the diagnostic criteria of tongue-tie, patient selection for early frenotomy has remained a challenge [6,14–18].

Previous studies have focused mostly on the infants. Several tongue-tie classifications with variable usability have been suggested, e.g., the Coryllos classification, the Hazelbaker assessment tool, the Kotlow classification, the Bristol Tongue Assessment Tool (BTAT), and the Neonatal Tongue Screening Test [19–22]. BTAT was further developed into a pictured tool, the picture assessment tool for tongue-tie in breastfed babies (TABBY) [23]. In a meta-analysis of 71 studies, ankyloglossia had been assessed using different tongue-tie severity scoring tools [8,19–22,24]. However, none of the previous scales included maternal breastfeeding symptom evaluation; the TABBY tool developers had highlighted the need for separate consideration of maternal symptoms as well [23]. Recently, a German version of the Bristol breastfeeding assessment tool, including a mother’s breastfeeding self-efficacy assessment, was published and validated [25].

Nowadays, the tongue-tie has turned out to be a globally problematic diagnosis. The clinical guideline for diagnosing neonatal oral surgical defects behind breastfeeding problems is lacking [6]. Despite of it, rapidly increased numbers of its interventions have been reported worldwide [13,26,27]. However, this does not reflect an increased burden of disease but rather changed diagnostic and treatment practices. Over-diagnostics and over-treatment have become problems, not the desired interests of the child, parents, or society. In New Zealand, for example, a multi-professional project was launched to support breastfeeding and reduce unnecessary frenotomies, reducing them by 7.8 percentage points in two years without negative impacts on breastfeeding [28].

**Fig 1:**
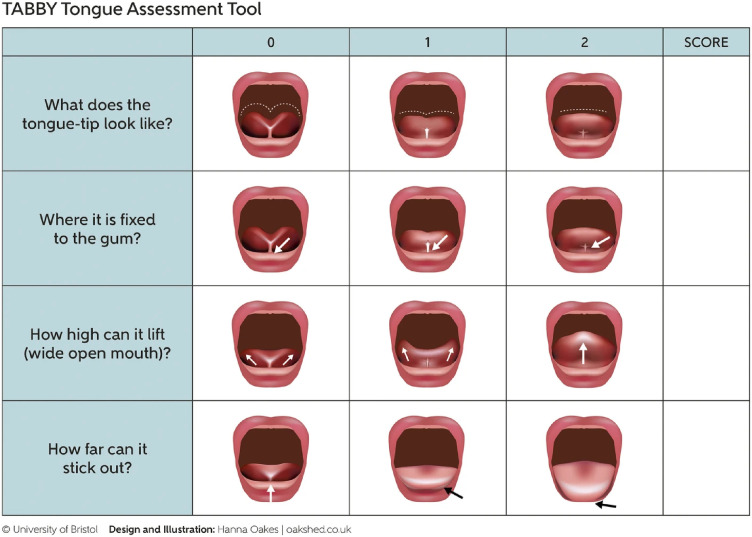
The picture assessment tool for tongue-tie in breastfed babies (TABBY) as presented in: Ingram J, Copeland M, Johnson D, Edmond A. Int Breastfeeding J 2019;14:31 [23]. In the present LINNE scoring, the TABBY scale was used upwards, i.e., the most difficult finding was given two points and the normal finding zero. Permission to reproduce the image is given publicly in the first page of the original publication.

The lingual frenulum in newborn infants (LINNE) project was launched to acquire the needed epidemiological data, improve the tongue-tie diagnostics, and to examine the efficacy and safety of tongue-tie therapies with adequate long-term follow-up protocols. In the present study, we aimed to prospectively validate the LINNE scoring in diagnosing newborns’ need for early tongue-tie treatments. It included three subscorings: the TABBY tool to evaluate the infant anatomy (Fig 1), a study group-developed maternal breastfeeding experience scoring (MBES) of acute breastfeeding symptoms, associated specifically to a tongue-tie (Table 1), and three anamnestic factors of the first-degree family members [23]. We hypothesized that the LINNE scoring would be reliable, including consistency in internal, inter-rater, and test-retesting over time, valid in terms of content and construct validities, accurate in diagnostics of a tongue-tie requiring early treatment, and feasible in the use of professionals treating neonates.

**Table 1:** Maternal breastfeeding experience questionnaire, the symptom scoring, and content validity analysis.

When I am breastfeeding my baby, I feel: (you may choose any of the below)	Score	Relevant, n*	Content validity index†
1. No problems in breastfeeding	0	14	1
2. Breastfeeding is painful	2	13	0.93
3. Baby has difficulties to grab the nipple	2	11	0.79
4. Baby needs bottle feeding	0.5	13	0.93
5. Baby has short feeding time	0.5	11	0.79
6. Baby gets tired or even falls asleep before finishing the meal, but wants to eat again soon	0.5	12	0.86
7. Baby has stomach problems	0.5	12	0.86
8. Baby has some other problems related to eating or breastfeeding	0.5	13	0.93

*Number of experts rating 4 or 5 (relevant/very relevant)

^†^Percent of the experts (n = 14) considering the item relevant (rating 4 or 5 on a scale 1–5)

## Methods

### Study design and participants

The present prospective validation study of the LINNE scoring, is a part of the LINNE project, registered in EudraCT (ID 2022-001546-37) and Clinicaltrials.gov (ID NCT05560750). The regional ethics committee of the Northern Ostrobothnia Hospital District (22.06.2022) and the administration of Oulu University Hospital, Oulu, Finland (22.09.2022), approved the LINNE research plan. All the parents gave written informed consent. The recruitment took place from October 3, 2022, to September 29, 2023.

Patients were identified and approached by the study and ward midwives in the Mother–child unit of Oulu University Hospital, Oulu, Finland. The study hospital provides specialized medical care within the public healthcare system in the Northern Ostrobothnia Well-being District, Northern Finland. All births in the area take place there. The Mother-child unit is the post-partum ward for all the families. All mother-infant-dyads are treated there, with or without medical conditions.

Families who expressed their interest were recruited and infants examined by the study physicians. Healthy, full-term newborns of healthy mothers were eligible until one month of age. The exclusion criteria were prematurity (gestation <37 + 0 weeks), serious illness requiring hospitalization of the mother and/or the newborn, infant’s major structural or chromosomal abnormality, and a non-breastfeeding mother. Otherwise, all neonates were welcome to the study. However, due to safety reasons, communication requirements of the trial follow-up, and methods of the ensuing logopedic follow-up, it was necessary that at least one of the caregivers was able to communicate in Finnish, at least marginally. Afterwards, at the six months’ age of exclusive breastfeeding milestone, the caregivers were e-mailed the follow-up Webropol questionnaire about the infant feeding maturation, and maternal breastfeeding experience.

The primary outcome of the study was the LINNE scoring’s validity in evaluating neonates’ early need for tongue-tie treatments. The LINNE scoring validation process is presented in Table 2. The secondary outcomes were the results of the LINNE subscorings’ validation tests.

**Table 2:** Process for validating the LINNE scoring.

LINNE scoring	Question	Statistical method
LINNE scoring, subscorings combined	Does the LINNE scoring detect the infants with tongue-ties needing early treatment?	
LINNE scoring reliability: internal consistency	Do all items of LINNE scoring assess the same phenomenon?	Cronbach alpha
1 TABBY tool	Do the individual or pooled TABBY scorings detect the tongue-ties needing early treatment?	AUC by ROC curves
1A Test-retest repeatability	Does the TABBY tool provide the same result on repeated assessments by differently educated healthcare professionals?	Fleiss’ kappa
2 Maternal breastfeeding experience scoring 2A Validity	Does MBES adequately measure the maternal breastfeeding experience?	
1 Content validity	Does MBES cover all symptoms commonly experienced by mothers breastfeeding an infant with a tongue-tie?	Expert survey
2 Criterion validity	Are the results of MBES comparable with a “gold standard”?	NA
3 Construct validity	Does MBES follow the theory on which it is based?	Hypotheses for convergent/discriminate validity
2B Responsiveness	Does MBES change over time or in response to treatment?	Effect size and standardized response mean
3 Anamneses of a tongue-tie among the study infants’ 1^st^ degree family members	Do the anamnestic factors predict the need for early tongue-tie treatment?	Sensitivity/specificity; positive/negative predictive values & likelihood ratios

LINNE, lingual frenulum in newborn infants; TABBY, picture tongue assessment tool for tongue-tie in breastfed babies; AUC, area under the curve; ROC, receiver operating characteristic curve; MBES, maternal breastfeeding experience scoring; NA, not applicable.

### Study tests and validation methods

The LINNE scoring, used in the study patient evaluation, consisted of three different scales: the TABBY tool, maternal breastfeeding experience, and anamnesis. These involved altogether 15 items that assessed infant tongue anatomy and function, mother’s perception of feeding, and some anamnestic features, previously linked to tongue-ties. Their scores were added together. The LINNE scoring range was 0–13. Infants with high LINNE scores (>8/13) were considered to need early tongue-tie therapy. To confirm that no infant was operated based on maternal symptoms/anamnesis only, at least four TABBY points were required. Therefore, the MBES and anamnesis scores were added together and maximally five points were counted to the LINNE scoring.

#### TABBY tool.

The TABBY tool was chosen to score the infant oral structures as it was considered practical and its interpretation clear (Fig 1) [23]. The TABBY tool elements consisted of the appearance and function of the tongue: A. the tongue tip shape/appearance, B. frenulum attachment underneath the tongue, C. elevation, and D. protrusion of the tongue. The scoring ranges of the pre-specified, individual TABBY elements were 0–2, and the pooled TABBY tool scores ranged 0–8. In contrast to the original TABBY study, and due to the other diagnostic features included in the LINNE scoring, the TABBY tool scorings were calculated upwards—that is, the most severe clinical manifestation gave the highest score, enabling the total LINNE scores to be summed up. Each infant’s two TABBY assessors—a physician and a midwife—were independent and mutually blinded: they completed their own research forms, not shown to each other. They examined the infants separately, and after completing, the forms were stored into the study files. The findings were not discussed until the examinations and interpretations were finished and recorded.

After one year of recruitment, we conducted an opinion survey to the TABBY tool assessors to examine its subjective usability. The survey included questions of whether the TABBY tool was helpful and easy to use as a test device for tongue-tie evaluation. The individual TABBY elements were rated on a 0–6 scale according to their clinical usability. The assessors’ professions and working experience in years were recorded as well.

#### Maternal breastfeeding experience.

The MBES consisted of eight questions in three subsets (Table 1). The first question aimed to detect asymptomatic breastfeeding mothers. The next two questions, on breastfeeding pain and infants’ difficulties in attaching/holding the nipple, were assumed to be the most specific ankyloglossia symptom scores [29,30]. The last five questions were designed to cover typical but non-specific symptoms involved in tongue-tie-caused breastfeeding problems.

The questionnaire formed a psychometric test, and its validation process included content and construct validities, and responsiveness testing. The questionnaire, based on literature review, was piloted by the study group physicians, and the item relevance, clarity, simplicity, and ambiguity were first judged in a group discussion. After revisions, content validity was tested by an invited expert group (n = 16), including neonatologists, speech therapists, and midwives. They rated the questionnaire by Likert scale 1–5 how well it covered the common maternal and infant symptoms when breastfeeding an infant with a tongue-tie. The item relevance was scored from 1 = not relevant, to 5 = very relevant, and content validity index was calculated by the number of experts providing a score of 4 or 5 divided by the total number of experts answered (Table 1). In the absence of a previous golden-standard test of maternal ankyloglossia-related breastfeeding symptoms for comparison, the criterion validity of MBES was not tested. For the construct validity testing, five hypotheses (constructs) were developed through consensus and tested (Table 3). Four hypotheses were included for the convergent evidence of construct validity and one hypothesis for the discriminate evidence of construct validity. The four convergent hypotheses indicated mothers without breastfeeding problems, mothers with specific tongue-tie symptoms, and mothers with milder, tongue-tie-associated breastfeeding problems. For the responsiveness testing, the six-month Webropol questionnaire responses on the breastfeeding success were used. The effect size ([post-treatment mean – pre-treatment mean]/standard deviation of the pre-treatment mean) and the standardized response mean ([post-treatment mean – pre-treatment mean]/standard deviation of the mean change score) were calculated [31]. In general, an effect size of >0.8 is considered a large effect.

**Table 3:** Hypotheses for the construct validity testing of the maternal breastfeeding experience scoring.

Hypotheses	Mothers who chose the alternatives, n	MBES, mean (SD)	p-value, a. vs. b.
1. Mothers whose infant had not severe tongue-tie would have chosen more often the alternative “no breastfeeding symptoms at all” than mothers whose infant had a tongue-tie needing early treatment			<0.001*
a. Infants without severe tongue-tie	128	.	
b. Infants with tongue-tie needing early treatment	2	.	
2. Mothers whose infant had not severe tongue-tie would have lower tongue-tie specific symptom scores than mothers whose infant had tongue-tie needing early treatment			<0.001^†^
a. Infants without severe tongue-tie	480	1.64 (1.43)	
b. Infants with tongue-tie needing early treatment	70	3.17 (1.15)	
3. Mothers whose infant had not severe tongue-tie would have lower tongue-tie associated symptom scores than mothers whose infant had tongue-tie needing early treatment			<0.001^†^
a. Infants without severe tongue-tie	477	0.56 (0.56)	
b. Infants with tongue-tie needing early treatment	69	1.05 (0.62)	
4. Mothers whose infant had not severe tongue-tie would have lower overall symptom scores than mothers whose infant had tongue-tie needing early treatment			<0.001^†^
a. Infants without severe tongue-tie	476	2.20 (1.64)	
b. Infants with tongue-tie needing early treatment	68	4.26 (1.21)	
5. Mothers of an infant with an even value LINNE study number would have more combined symptom scores than mothers of an infant with an odd value LINNE study number			0.914^†^^‡^
a. Infants with an even value LINNE study number	274	2.44 (1.68)	
b. Infants with an odd value LINNE study number	272	2.46 (1.79)	

*Pearson Chi-square test, ^†^Two-tailed independent samples t-test, ^‡^No significant difference for discriminate hypothesis.

MBES, maternal breastfeeding experience scoring; LINNE, lingual frenulum in newborn infants.

#### Anamnestic factors.

Tongue-tie problems may reside in some families. To study the effect of the previous tongue-tie-related problems in the family, we asked about previous breastfeeding problems, tongue-tie surgery, and speech errors among the first-degree family members (i.e., mother, father, and siblings). When the anamnesis was positive, one point was given, otherwise none. The accuracy studies for the diagnostic tests, such as sensitivity, specificity, positive and negative predictive values, and positive and negative likelihood ratios, were calculated with 95% confidence intervals (CI).

### Sample and effect size

The sample size of the present prospective validation study was determined by the concomitant LINNE treatment trial pilot study. The validation study patient data collection continued until the treatment trial pilot reached its calculated sample size, the prospectively set primary outcomes were established at one-month controls, and the completion of their analyses in October 2023. The sample size of the present observation study was then n = 556. The clinical meaningfulness of the reached sample size was estimated calculating the study effect size. With the alpha error rate 0.05 and 80% power, the present study effect size was 0.825, which is generally considered large effect size [32].

#### Data management procedures.

Case record files were entered to an Excel file, situated in the electronic research database of the study hospital m-drive, accessible only to study team members. The data of the participants was collected from the completed study forms and Webropol questionnaires. Only information necessary for the study was stored in the database. All paper documents are being kept in the principal investigator’s closed office until the official storage period of the research archive has ended. Only the study personnel will know the identity and other identifiable information of the study patients, and they are all bound by secrecy. However, an inspector external to the study (e.g., monitor) has the right to revise the study documents, if necessary. A self-assessment of the security risks of the study data has been stated, as appropriate, and stored for parental access upon demand.

The data quality has been ensured by data organization and documentation practices, concerning all study members. Upon recording to the Excel file, all data has been double-checked. The encountered inconsistencies have been documented and corrected. As the recruitment proceeded, small subsets have been routinely inspected. Data validation tools of SPSS Statistics, such as outlier detection, format checks, range checks, and consistency checks, have been used.

### Statistical analysis

All statistical analyses were performed using IBM SPSS Statistics (IBM Finland Ab, Helsinki, Finland) version 29.0.1.0. The LINNE scorings’ internal consistency, reflecting reliability, was validated by Cronbach’s alpha; values >0.70 were considered acceptable. The analysis included 15 items: four TABBY scores, eight MBES scores, and three anamnestic factors.

To analyze the efficacy of the pre-specified pooled and individual TABBY elements in diagnosing neonates’ early need for tongue-tie therapy, we used receiver operator characteristic curves (ROC) with the area under the curve (AUC) calculations of all raters, and separately for physicians and midwives. To examine the variability in the diagnostic accuracy of pooled and individual TABBY scorings between two independent observers, we used Fleiss’ multi-rater kappa (κ) analysis [33].

The dichotomous values were analyzed using the Pearson Chi-square test. Non-parametric data were examined using the Mann-Whitney U test or Spearman rank correlation. Continuous variables were analyzed using the two-tailed independent samples t-test and the Pearson correlation, as appropriate. Significance was set to p < 0.05.

## Results

**Fig 2:**
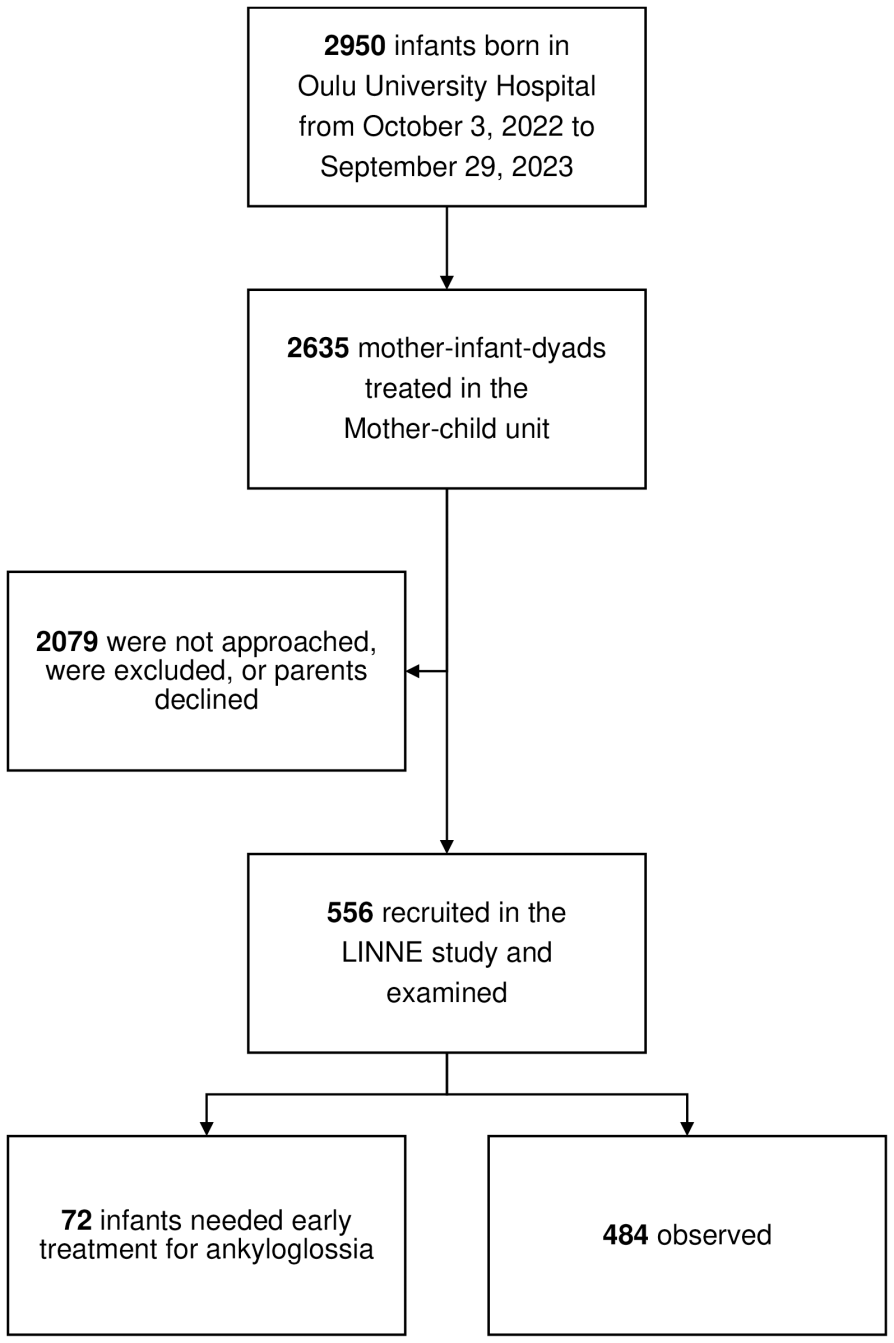
Flow chart of the study patients. LINNE, lingual frenulum in newborn infants.

Between October 3, 2022 and September 29, 2023, eligible mother-infant-dyads were recruited in the Mother–child unit of Oulu University Hospital, Oulu, Finland (Fig 2, Table 4). During that time, of the total 2950 inborn infants, 2635 were treated in the Mother–child unit. Of them, 2079 infants were not included: as recruitment took place generally during the office hours, most of them were not approached, parents did not express their willingness to participate, or exclusion criteria were met. Altogether 556 infants (18.8%) entered the LINNE observation study and were assessed by the study physicians (n = 5) and ward midwives (n = 82; Table 3). Overall, 1094 TABBY tool evaluations were performed. All 556 study mothers completed the MBES questionnaire. Seventy two (12.9%) infants met the LINNE treatment trial inclusion criteria. Performing the tests did not cause any adverse events, complications, pain, or severe discomfort to the infants. Combining the three subscorings’ results, the LINNE scoring’s internal consistency, Cronbach α = 0.723, was acceptable.

**Table 4:** Characteristics of the study infants.

	Observed infants, n = 484	Treated infants, n = 72	p-value
Gestational age at birth, mean (SD), weeks	39.5 (1.9)	39.3 (1.2)	0.483*
Birth weight, mean (SD), g	3593 (437)	3611 (526)	0.754*
Postnatal age at study entry, median (IQR), d	2.0 (1)	2.0 (2)	0.130^†^
Maximal weight decrease, % of birth weight, mean (SD)	6.3 (2.4)	5.7 (3.5)	0.120*
Females, n (%)	239 (49.4)	18 (25.0)	<0.001^‡^
Multiple gestation, n (%)	3 (0.6)	3 (4.2)	0.007^‡^
1 min Apgar score, median (IQR)	9 (0)	9 (0)	0.439^†^
5 min Apgar score, median (IQR)	9 (0)	9 (1)	0.647^†^
Umbilical arterial pH, mean (SD)	7.24 (0.3)	7.26 (0.1)	0.458*
Umbilical arterial base excess, mean (SD)	−5.1 (3.6)	−4.7 (3.0)	0.444*
Maternal any medication during pregnancy, n (%)	309 (63.8)	39 (54.2)	0.113^‡^
Phototherapy, n (%)	101 (20.9)	14 (19.4)	0.781^‡^
Hypoglycaemia (blood glucose <3), during ward stay, n (%)	43 (8.9)	11 (15.7)	0.178^‡^
Maternal type 1, 2, or gestational diabetes, n (%)	89 (18.4)	10 (13.9)	0.352^‡^
Previous breastfeeding experience, n (%)	258 (52.0)	46 (63.9)	0.107^‡^
Previous breastfeeding problems, n (%)	99 (20.5)	33 (45.8)	<0.001^‡^
First-degree family member with a previous frenotomy, n (%)	60 (12.4)	33 (45.8)	<0.001^‡^
First-degree family member with a previous articulation error, n (%)	69 (14.3)	27 (38.9)	<0.001^‡^

*Two-tailed independent samples t-test, ^†^Mann-Whitney U test, ^‡^Pearson Chi-square test.

### TABBY tool diagnostics

The pooled and individual (A–D) infant TABBY scores of the observed vs. treated infants differed significantly (Table 5). The pooled TABBY scorings’ AUC (95% CI) = 0.914 (0.891–0.938) for detecting tongue-tie requiring therapy. The physicians’ pooled TABBY AUC = 0.948 (0.924–0.973) and the midwives’ 0.870 (0.830–0.911) for early tongue-tie treatment. The pooled scores did not differ, but tongue tip shape (A, p = 0.043) and frenulum fixation (B, p = 0.041) in the subscorings did. The combined results suggest that TABBY tool was usable for both physicians and midwives; the differences in the subscales may refer to variances of experience in investigating the neonates.

**Table 5:** TABBY tool scores and the AUC-values of the pooled and individual (A-D) elements by study physicians and midwives, inter-rater agreements by Fleiss multi-rater kappa values, and TABBY scores of the study infants. Assessing physicians n = 5, midwives n = 82.

	All examinations (n = 1094)		Physicians’ examinations (n = 559)		Midwives’ examinations (n = 535)		Physicians vs. Midwives			TABBY scores				
										a. Observed infants (n = 484)		b. Treated infants (n = 72)		a. vs. b.
	Md (IQR)	AUC (95% CI)	Md (IQR)	AUC (95% CI)	Md (IQR)	AUC (95% CI)	p- value*	Fleiss κ (95% CI)	Fleissκ p-value	Mean (SD)	Md (IQR)	Mean (SD)	Md (IQR)	p- value*
TABBY scores, pooled	1.0 (3)	0.914 (0.891-0.938)	1.0 (3)	0.948 (0.924-0.973)	1.0 (3)	0.870 (0.830-0.911)	0.751	0.441 (0.406-0.477)	<0.001	1.43 (1.60)	1.0 (2)	4.91 (1.77)	5.0 (2)	<0.001
A Tongue tip shape	0 (1)	0.832 (0.794-0.870)	0 (1)	0.858 (0.809-0.907)	0 (1)	0.796 (0.737-0.855)	0.043	0.473 (0.403-0.544)	<0.001	0.29 (0.50)	0 (1)	1.19 (0.66)	1.0 (1)	<0.001
B Frenulum fixation	0 (1)	0.850 (0.815-0.885)	0 (1)	0.895 (0.859-0.931)	0 (1)	0.807 (0.750-0.864)	0.041	0.504 (0.436-0.572)	<0.001	0.34 (0.54)	0 (1)	1.34 (0.64)	1.0 (1)	<0.001
C Tongue elevation	0 (1)	0.819 (0.780-0.857)	0 (1)	0.862 (0.820-0.904)	0 (1)	0.775 (0.712-0.838)	0.152	0.402 (0.339-0.476)	<0.001	0.32 (0.50)	0 (1)	1.15 (0.63)	1.0 (1)	<0.001
D Tongue protrusion	1.0 (1)	0.803 (0.769-0.837)	1.0 (1)	0.810 (0.763-0.858)	1.0 (1)	0.788 (0.738-0.839)	0.328	0.351 (0.279-0.423)	<0.001	0.48 (0.56)	0 (1)	1.24 (0.52)	1.0 (1)	<0.001

*Mann-Whitney U test.

TABBY, picture assessment tool for tongue-tie in breastfed babies; Md, median; AUC, area under the curve; CI, confidence interval; κ, kappa.

We analyzed, by determining the Fleiss’ kappa, the agreement between two independent observers’ diagnostic pooled and TABBY subscorings (Table 5). Within both groups, examinators varied day to day, depending on the staff situation. The pooled TABBY scores’ κ (95% CI) = 0.441 (0.406–0.477), showing moderate agreement between physicians and midwives. Three subscorings (A-C) showed moderate strength of agreement; the best was detected for frenulum fixation (B; κ = 0.504, 0.403–0.544). Although tongue protrusion showed the lowest (fair) agreement (D; κ = 0.351, 0.279–0.423), the confidence interval upper border reached the moderate agreement level. However, all the determined kappa values were statistically significantly different from zero (p < 0.001; Table 5).

### TABBY user opinion survey

All five study physicians and 32 (39%) midwives responded to the opinion survey (Table 6). TABBY tool usability and diagnostic value reached the adequate level: 60% of the physicians and 43% of the midwives considered the TABBY tool easy to use. 60% of the physicians and 50% of the midwives found it very useful in evaluating the frenulum. Two (6.7%) midwives found it not useful. The opinion associations between the two groups were: 1. TABBY usability,χ^2^ = 3.447, p = 0.178; 2. Scoring easiness, χ^2^ = 0.938, p = 0.816; 3. TABBY usefulness in the frenulum evaluation, χ^2^ = 0.682, p = 0.711. No correlation between the respondents’ opinions and working experience (years) emerged: TABBY usability ρ = −0.024, p = 0.891; scoring easiness ρ = 0.018, p = 0.918; TABBY usefulness ρ = −0.119, p = 0.498.

**Table 6:** Survey of the TABBY tool user opinions. Respondent numbers: physicians n = 5, midwives n = 32.

Survey questions	Agree		Neutral		Disagree		Strongly disagree	
	Physicians	Midwives	Physicians	Midwives	Physicians	Midwives	Physicians	Midwives
TABBY form was easy to use, n (%)	5 (100)	17 (57)	0	12 (40)	0	1 (3)	0	0
TABBY tool scoring was easy to do, n (%)	3 (60)	13 (43)	2 (40)	13 (43)	0	3 (10)	0	1 (3)
TABBY tool was useful in frenulum evaluation, n (%)	3 (60)	13 (43)	2 (40)	15 (50)	0	2 (7)	0	0

Usability of TABBY tool individual elements, score	Physicians	Midwives	p-value*					
A. Tongue tip appearance, median (IQR)	5.0 (0)	5.0 (2)	0.350					
B. Frenulum fixation point to the tongue, median (IQR)	5.0 (2)	5.0 (2)	0.958					
C. Tongue elevation, median (IQR)	5.0 (2)	4.5 (2)	0.980					
D. Tongue protrusion, median (IQR)	4.0 (1)	4.0 (2)	0.738					

*Mann-Whitney U test

TABBY, picture assessment tool for tongue-tie in breastfed babies.

### Maternal breastfeeding experience scoring

For the content validity testing, 14/16 (88%) experts evaluated the MBES. The relevance of each item was separately asked and content validity indexes calculated (Table 1). MBES was found to cover all important symptoms of mothers breastfeeding an infant with tongue-tie. On a scale of 0–5, median (IQR) content validity score was 4.0 (1.0).

In the construct validity testing, all four convergent hypotheses were significant (p < 0.001; Table 3). One discriminate hypothesis showed no significant difference between the two groups (p = 0.14).

For the responsiveness validation, 256 (46%) caregivers returned the six-month follow-up questionnaire. The observed infants’ MBES median (IQR) was 2.5 (2.5) at study onset, and 2.5 (2.5) after six months. In the tongue-tie treated, the MBES median (IQR) changed from 4.3 (2.0) to 2.7 (1.75). The overall calculated effect size was 1.57, and the standardized response mean for the whole MBES was 0.83. The low respondent percentage may have caused a non-responder bias. To understand the possible bias, all available mother-infant-dyad data, i.e., prenatal, delivery-associated, and postnatal variables were analyzed, comparing respondents vs. non-respondents. The following significant differences were found: the respondents’ infants were born at lower (mean [SD]) gestational age (39.3 [2.3] vs. 39.6 [1.3] wk, p = 0.018), had smaller (mean [SD]) birth weights (3545 [468] vs. 3637 [429] g, p = 0.015), and had higher LINNE (mean [SD]) scores (5.0 [2.9] vs. 4.3 [3.0], p = 0.006) vs. the non-respondents’ infants (two-tailed independent samples t-test). The responded mothers had had fewer previous pregnancies (n, median [IQR]) vs. non-responders (2 [2] vs. 2 [3], p = 0.007, Mann-Whitney U test), and had more often other than pregnancy-related diseases (n = 63 (25.6%) vs. n = 51 (17.3%), p = 0.019, Chi-square test). No other differences were revealed.

### Family anamnesis

The frequencies of the positive family anamneses ranged 12.6–50.8%, depending on the study group, with significant differences between the treated vs. observed infants (Table 7). In determining the need for early tongue-tie treatment, sensitivities ranged 23.7–33.7% and specificities 90.6–91.7%, with alleged positive and negative predictive values and likelihood ratios (Table 7).

**Table 7:** Validity of the LINNE scorings’ family anamnestic factors.

Anamnestic factor (respondents, n)	Positive anamnesis in treated vs. observed infants, n (%)	Observed vs. treated, p*-*value*	Sensitivity (95% CI)	Specificity (95% CI)	Positive predictive value (95% CI)	Negative predictive value (95% CI)	Positive likelihood ratio (95% CI)	Negative likelihood ratio (95% CI)
Previous breastfeeding problems (n = 493)	33 (45.8) vs. 99 (20.5)	<0.001	23.7% (16.7-31.9)	91.7% (88.4-94.3)	50.8% (37.7-63.9)	76.8% (72.6-80.6)	2.86 (1.80-4.49)	0.83 (0.74-0.91)
Tongue-tie operated in the first-degree family members (n = 549)	33 (45.8) vs. 60 (12.4)	<0.001	33.7% (24.2-44.3)	91.5% (88.5-93.9)	44.3% (32.4-56.7)	87.3% (83.9-90.1)	3.95 (2.59-5.92)	0.72 (0.61-0.82)
Tongue-tie-related pronunciation errors in the first-degree family members (n = 553)	28 (38.9) vs. 69 (14.3)	<0.001	28.1% (19.4-38.2)	90.6% (87.5-93.1)	38.6% (27.2-51.0)	85.7% (82.3-88.7)	2.99 (1.94-4.53)	0.79 (0.68-0.89)

*Pearson Chi-square test.

LINNE, lingual frenulum in newborn infants; CI, confidence interval.

## Discussion

The present validation study demonstrated that the LINNE scoring accurately detected the need for early tongue-tie treatments in newborn infants. Its internal consistency was high with Cronbach alpha 0.723, combining all three subscorings. The TABBY tool had AUC 0.914, good test–retest repeatability, and usability in the opinion survey. MBES reached the required content and construct validities and responsiveness. The family anamnesis was highly specific but had fair sensitivity.

For the LINNE scoring, combining the three different subscales demanded prior estimations of their relative impacts upon the study planning. In the absence of previous maternal symptom data-based scoring threshold, we had to adhere to the clinical reasoning. The study group decision was that no infant should be operated without obvious tongue-tie findings. This was ensured by setting the operative treatment threshold level high enough (>8). According to the preliminary calculations, this required at least four TABBY points. The combination of the two other subscales were therefore restricted up to five points. This worked well; no infant was referred to the treatment trial without actual tongue-tie.

The pooled TABBY tool scorings detected the tongue-ties needing early treatment (Table 5). Previously, the TABBY tool was found to be suitable for midwives’ and physicians’ use [23]. The present AUC values of the pooled TABBY scores did not differ between the two groups of assessors (Table 5), implicating test–retest reliability. Two TABBY elements (A, B) reached statistical significance between the assessor groups that could have reflected differences in experience. Using Fleiss’ kappa analysis, we evaluated the inter-rater agreement of the two assessors from an assembly of multiple independent raters. The pooled TABBY scorings represented a moderate level of agreement, which is acceptable for clinical use. TABBY element D (tongue protrusion) showed only fair agreement, but more importantly, the 95% confidence interval upper limit reached the moderate agreement level. The Fleiss’ kappa p-values were highly significant, suggesting that the agreements pointed indeed to the same direction (Table 5). We found some infants’ cooperation varying daily, possibly hindering the assessment, limiting test repeatability, and even fluctuating the results. For the most accurate results, we recommend using TABBY tool assessments for alert infants who are just starting to eat. In the statistical analysis of the TABBY opinion survey, no correlations were found between the TABBY scores and work experience.

For approaching the maternal side, we developed a psychometric questionnaire to evaluate acute maternal breastfeeding symptoms specifically associated to tongue-tie. MBES covers breastfeeding painfulness, an infant’s inability to latch, and a series of different symptoms that have been clinically used as signs of a tongue-tie. We analyzed its content and construct validities and responsiveness. The content validity of MBES was given good feedback in the expert survey*.* An alternative to the expert survey could have been an analysis of the floor and ceiling effects. However, it was not used due to the very short test scales used (0–0.5 or 0–2). We did not include large scales or numerous questions, as the need for practicality in clinical use was preferred over inclusivity. The construct validity of MBES was verified, as all four convergent hypotheses differed significantly while the discriminate hypothesis did not (Table 3). After six months, MBES changed from 4.3 to 2.7 in the group of treated infants, whereas no change was found in infants without tongue-tie. For the quantification of responsiveness and the magnitude of the treatment effect, the effect size and standardized response mean are commonly used. The MBES scorings’ effect size was 1.57 and the standardized response mean 0.83, suggesting large effect magnitudes and acceptable responsiveness. However, the low respondent percentage may have caused non-responder bias. To understand better the impact of the potential bias, we analyzed the respondent and non-respondent baseline data. Although some differences were revealed, we found only the respondents’ higher LINNE scorings clinically meaningful. They could explain the better motivation of the parents to answer to the later inquiries, not compromising the validity of the MBES. The criterion validity was not tested due to the absence of a previous golden-standard scoring that would have focused on the tongue-tie-caused maternal breastfeeding symptoms. Nevertheless, the reliability and validity results encouraged the continued use of MBES scoring.

Tongue-tie inheritance or family member incidence have not been studied before. Therefore, three anamnestic factors that could predispose infants to tongue-tie breastfeeding problems were included. The significant differences between the two groups’ anamnestic factors suggest that they may have role in the tongue-tie diagnostics (Table 7). All LINNE scorings of the three anamnestic factors of first-degree family members showed high specificity (>90%) in determining the need for early tongue-tie treatments, while their sensitivities were 30% (Table 7). Low sensitivity can be explained by the diverse etiologies of speech errors and the limited part of tongue-tie among them. High specificity of the family member tongue-tie operations refers to positive inheritance, deserving further research. These findings provide additional information about tongue-tie incidences and risks in infants with a positive family anamnesis. We found these results helpful in evaluating and approaching the potential anatomical faults.

### Strengths and limitations

Our patients represent a non-selected, heterogenous group. The study hospital serves all the laboring families in the area, and no patient selection was included, except the exclusion criteria, based on morbidity. We find our research highly generalizable. Another strength of this study is the sample size of 556 infants, 1094 TABBY scorings accomplished, with large overall effect size (0.825). The TABBY tool was tested in both midwives’ and physicians’ use. Mostly highly specialized study doctors (4/5) and even newly started young midwives, without standardized guidance, showed no large difference in the results, reflecting good reliability. In the statistical analysis of the user opinion survey, no correlations were found between the different work experiences. Therefore, the TABBY tool was found to be a suitable screening tool in use of healthcare professionals, although tongue-tie diagnostics require considering the maternal symptoms as well.

The present study has some limitations. Although no patient selection was conducted, the impact of cultural breastfeeding behaviors was not completely excluded. These differences may prevail, but as breastfeeding is internationally recommended and great interest of most of the families, their role remains open, regarding the tongue-tie results. The variations of human macro anatomy may be between individuals or depend on ethnicity as well. Further studies in different cultures are required. The study physicians’ number was low compared with the midwives, which may have affected the inter-rater agreement results. Some limitations were found in the daily TABBY tool use, as some TABBY scorings of a patient may have varied. Moreover, the infants’ recruitment age was not standardized in the methods, although the limit was set to one month of age. However, the recruitment took place during the first postnatal days in most cases (Table 4). The recall bias concerning family anamnesis is not completely excluded either. However, parents remembered well, if their older child had had a tongue-tie operation, articulation errors, or remarkable feeding problems in the beginning. We also found that many adults today are informed and aware of their own previous tongue-tie operations, if they have been performed in the childhood. They usually remember the possible speech rehearsal sessions with logopedics during childhood as well. It is likely however that some recall bias has not been overcome.

## Conclusion

The LINNE scoring was confirmed to be a valid diagnostic tool for a tongue-tie in neonatal research use and a practical assessment tool for healthcare professionals. Further studies, testing among other nations and cultures, physician training impact on the TABBY executions and inter-rater agreements, validating the scoring in premature infants and neonatal intensive care settings, the tongue-tie epidemiology, and the efficacy and safety of its therapies, are justified.
